# Metabolomic Profiling to Identify Predictors of Response to Vitamin E for Non-Alcoholic Steatohepatitis (NASH)

**DOI:** 10.1371/journal.pone.0044106

**Published:** 2012-09-19

**Authors:** Jianfeng Cheng, Andrew Joyce, Katherine Yates, Bradley Aouizerat, Arun J. Sanyal

**Affiliations:** 1 Division of Gastroenterology, Hepatology and Nutrition, Department of Internal Medicine, Virginia Commonwealth University School of Medicine, Richmond, Virginia, United States of America; 2 Venebio Group, LLC, Virginia BioTechnology Research Park, Richmond, Virginia, United States of America; 3 Johns Hopkins Bloomberg School of Public Health, Baltimore, Maryland, United States of America; 4 Department of Physiological Nursing, University of California San Francisco, San Francisco, California, United States of America; Institute of Hepatology London, United Kingdom

## Abstract

Vitamin E was recently shown to improve hepatic histology in a randomized controlled trial of pioglitazone or vitamin E for nonalcoholic steatohepatitis (PIVENS). The current study utilized samples collected in the PIVENS trial to identify: (1) baseline metabolomic profiles that could identify who would respond to vitamin E treatment and (2) end of treatment metabolomic profiles reflective of histologic improvement. A comprehensive analysis of metabolomics profiles (n = 547) quantified by mass spectrometry was performed in vitamin E responders (n = 16), vitamin E non-responders (n = 15), and placebo responders (n = 15). At baseline, phenyl-propionic acid (Odds ratio: 29.4, p<0.01), indole-propionic acid levels (Odds ratio: 16.2, p<0.01) were directly associated with a subsequent histologic response to vitamin E treatment whereas γ-carboxyethylhydroxychroman (CEHC) levels were inversely related to histologic response. Adjusting for baseline values by analysis of covariance, the end of treatment levels of gamma-glutamyl leucine (Fold change: 0.82, p<0.02) and gamma-glutamyl valine (Fold change: 0.8, p<0.03) were significantly lower in vitamin E responders compared to non-responders. The levels of gamma-glutamyl transpeptidase were not significantly different across the two groups. Subjects receiving placebo who demonstrated a histologic improvement also demonstrated lower levels of gamma-glutamylated amino acids (leucine, valine and isoleucine) compared to vitamin E non-responders. These data provide exploratory proof that there are measurable differences in the metabolic profile of subjects who are likely (vs unlikely) to respond to vitamin E treatment for NASH and in those experiencing histologic improvement (vs no improvement) on treatment and support further studies to validate these biomarkers.

## Introduction

Nonalcoholic steatohepatitis (NASH) is a common liver disease that is characterized by predominantly macrovesicular hepatic steatosis, hepatocellular ballooning and lobular inflammation often with a centrilobular distribution [1], [2]. NASH affects 4–5% of the US population and can progress to cirrhosis in up to 15% of subjects [3], [4].

Insulin resistance and oxidative stress have been implicated as two important pathophysiologic factors that cause NASH [5]–[8]. The “Pioglitazone versus Vitamin E versus Placebo for the Treatment of Nondiabetic Patients with Nonalcoholic Steatohepatitis” (PIVENS, ClinicalTrials.gov number, NCT00063622.) was a multicenter, prospective, placebo-controlled clinical trial in non-diabetic, non-cirrhotic subjects with histologically-active NASH that targeted insulin resistance with pioglitazone (30 mg/day) or oxidative stress with vitamin E (RRR- α-tocopherol 800 IU/day) [9]. In this trial, 43% of subjects receiving vitamin E met the primary endpoint of histologic improvement compared to 19% of those receiving placebo (p<0.001) [9]. These data provide hope for effective pharmacologic therapy for NASH.

Given that only some patients with NASH respond to vitamin E treatment, there is a need to develop methods to identify such individuals prior to starting therapy. Also, once treatment is started, one has to determine if a given individual is responding to treatment. In the PIVENS trial, the aspartate aminotransferase (AST) and alanine aminotransferase (ALT) declined in subjects receiving vitamin E but did not reliably predict histologic improvement [9]. A liver biopsy is thus needed to determine if a given subject is responding to treatment; however, liver biopsies are invasive, uncomfortable and occasionally associated with clinically significant morbidity and mortality [10], [11]. Therefore, a need to develop non-invasive methods to determine the presence of an on-treatment response exists.

Metabolomic technologies allow measurement of a multitude of metabolites in a single plasma or tissue sample. An advantage of analyzing metabolomic datasets is that they can be used to identify unique “metabolic signatures” of disease states in circulation. They also provide an unbiased approach to identification of such signatures. These considerations have led to their utilization in biomarker development.

The objectives of this pilot exploratory study were to utilize the plasma samples collected during the PIVENS trial to determine whether there were significant measurable differences in the metabolomic profiles of subjects who did or did not respond to vitamin E treatment at baseline and at end of treatment. The goal was to generate pilot data to provide direction for future focused large scale studies to develop: (1) baseline predictors of histologic response to vitamin E and (2) biomarkers indicative of histologic response to vitamin E.

## Materials and Methods

This study was approved by NASH CRN Steering Committee (SC). It was conceived as an ancillary study to the PIVENS trial that was performed by the NASH Clinical Research Network of the National Institutes of Diabetes, Digestive and Kidney Diseases (NIDDK). The protocol was approved by the ancillary studies committee and supported via an ARRA supplement to the institutional contract for the NASH CRN to Virginia Commonwealth University (VCU). All participants involved in this ancillary study provided written consent to donate serum/plasma samples for the main study objectives and for future NASH CRN ancillary studies. The study was considered exempt from a separate formal institutional review because it involved retrospective analysis of samples collected during the study. Also, the scope of the analyses was covered under the original IRB approval for the study at all sites. The data were analyzed by the investigators and the manuscript prepared entirely by the investigators.

### Patients and samples

The PIVENS trial was conducted by the NIDDK NASH CRN from 2005 to 2008. The details of the study protocol and the main results of the trial have already been published [9], [12]. Briefly, non-diabetic, non-cirrhotic adults with active NASH were enrolled. Active NASH was defined by the presence of definite or possible steatohepatitis with a NAFLD activity score (NAS)≥5 as assessed by the CRN site pathologist or definite steatohepatitis with a NAS≥4 as assessed by a CRN site-pathologist and another NASH CRN pathologist [9], [12]. Subjects who had a liver biopsy within the previous six months were randomized to receive 96 weeks of treatment with one of three treatments: (1) pioglitazone (30 mg once a day by mouth) and vitamin E-placebo (n = 80), (2) vitamin E (800 IU once a day by mouth) and pioglitazone-placebo (n = 84) and (3) vitamin E-placebo and pioglitazone-placebo (n = 83). A liver biopsy was performed after 96 weeks of treatment and the treatments were then discontinued. 90% of subjects had both a baseline and an end of treatment biopsy.

For the purpose of this analysis, response was defined by a decrease in the NAS by two or more points with at least one point improvement in hepatocyte ballooning. This is identical to the primary endpoint of the PIVENS trial [9]. Non-response was defined as not meeting the primary outcome of the trial, no resolution of steatohepatitis (as determined by NASH CRN central pathology review) and ballooning score greater than zero. This was done to maximize the ability to identify differences in analytes by studying the extreme ends of the spectrum of histologic response in the trial.

Thirty six of the 84 subjects who received vitamin E met the primary endpoint; of these, 16 subjects were selected (group 1). This was based on the availability of paired plasma samples and also histologic data at end of study for analysis. For the metabolomic analyses, 15 subjects each from two control groups: (1) vitamin E nonresponders, and (2) placebo responders. The rationale for the former group was that it was the primary group for comparison while that for the latter group was to assess the specificity of potential analytes for response to vitamin E treatment. A stratified random sampling scheme was used to select the samples. The strata were determined by the subjects meeting each group's inclusion criteria and the availability of pairs of liver biopsy tissue and fasting plasma specimens for these subjects. For the vitamin E non-responder group, 15 of the 36 subjects who met the definition of non-response (group 2) were randomly selected. The placebo responder group included 15 of the 16 subjects in the placebo arm who had a histologic response (group 3).

### High-throughput metabolomic profiling

All samples were processed by Metabolon (Research Triangle Park, NC) using GC/MS and LC/MS/MS platforms with the methodological details summarized as follows:

#### Sample Preparation

Upon receipt of plasma samples, aliquots were immediately stored at −80°C until time of analysis when samples were extracted and prepared for analysis using Metabolon's standard solvent extraction method. The extracted samples were split into equal parts for analysis on the GC/MS and LC/MS/MS platforms. Also included were several technical replicate samples created from a homogeneous pool containing a small amount of all study samples (“Client Matrix”).

#### Liquid chromatography/Mass Spectrometry (LC/MS, LC/MS2) analysis

The sample extract for LC/MS/MS was split into two aliquots, dried, then reconstituted in acidic or basic LC-compatible solvents, each of which contained 11 or more injection standards at fixed concentrations. One aliquot was analyzed using acidic positive ion optimized conditions and the other using basic negative ion optimized conditions in two independent injections using separate dedicated columns. The MS analysis alternated between MS and data-dependent MS^2^ scans using dynamic exclusion.

#### Gas chromatography/Mass Spectrometry (GC/MS) analysis

GC/MS sample extracts were re-dried under vacuum desiccation for a minimum of 24 hours prior to being derivatized under dried nitrogen using bistrimethyl-silyl-triflouroacetamide (BSTFA). The GC column was 5% phenyl and the temperature ramp is from 40° to 300°C in a 16 minute period. Samples were analyzed on a Thermo-Finnigan Trace DSQ fast-scanning single-quadrupole mass spectrometer using electron impact ionization. The instrument was tuned and calibrated for mass resolution and mass accuracy on a daily basis.

#### Metabolite identification

Compounds were identified by comparison to library entries of purified standards or recurrent unknown entities. Identification of known chemical entities was based on comparison to metabolomic library entries of more than 2362 commercially available purified standards and additional presently unknown entities were identified by virtue of their recurrent nature.

#### Data normalization

Samples were analyzed over the course of two weeks. Each run day was balanced for different sets with samples completely randomized to avoid group block effects. For example, a random selection of samples was made on each run day choosing the same number from each group – say 3 vitamin E responders, 3 vitamin E non-responders, and 3 placebo responders. Additionally, metabolites in each sample were normalized by dividing respective metabolite raw area counts by their median value for each run-day to correct for variation resulting from instrument inter-day tuning differences. Missing values were assumed to result from areas falling below limits of detection. As such, missing values were imputed with the observed minimum for each metabolite after the normalization step.

### Statistical analysis

Statistical analyses were performed in the R statistical computing environment (http://cran.r-project.org/) and SAS (V 9.2, Cary, NC). Categorical data were analyzed by chi-square test. Analysis of variance (ANOVA) and analysis of covariance (ANCOVA) models were developed to analyze continuous variables followed by Tukey post-tests for multiple comparisons to identify specific differences between groups. Given the small sample size which precluded meaningful multiple comparisons analyses for the number of analytes (n = 547), a smaller set of analytes that were significant on univariate analysis was included in a logistic regression model to identify those that predicted histologic outcomes of vitamin E treatment.

## Results

### Study Population

A total of 46 pairs of fasting plasma samples obtained at baseline and end of treatment were analyzed in vitamin E responders (n = 16), vitamin E non-responders (n = 15) and placebo-responders (n = 15). **Table 1** summarizes the baseline demographic, clinical, laboratory and histologic data from these three groups. The three groups were comparable with respect to age, gender, race, liver enzyme values, distribution of specific features of the metabolic syndrome, severity of underlying insulin resistance and histologic features of NASH. Specifically, the severity of steatosis, inflammation, hepatocyte ballooning and fibrosis were all comparable across the groups.

**Table 1:** Baseline characteristics of study population.

Characteristic	Vit.E responder(n = 16)	Vit.E non-responder (n = 15)	Placebo responder (n = 15)	p-value
**Demographic factors**				
age(yr)	43.6±12.9	48.1±6.4	44.4±8.3	ns
female sex (%)	62.5	73.3	46.7	ns
Race or ethnic group (%)				
non-hispanic white	53.3	73.3	60	ns
non-hispanic other	20	13.3	20	
any Hispanic	26.7	13.3	20	
**Serum biochemical levels**				
Alanine aminotransferase (U/liter)	97±45	106±59	73±40	ns
Aspartate aminotransferase (U/liter)	69±39	70±42	46±23	ns
γ-Glutamyltransferase (U/liter)	60±42	47±23	67±73	ns
Alkaline phosphatase (U/liter)	72±17	92±28	80±25	ns
Total bilirubin (mg/dl)	0.87±0.37	0.72±0.31	0.77±0.45	ns
**Lipids**				
Triglycerides (mg/dl)	142±103	197±110	156±79	ns
Cholesterol (mg/dl)				
Total	189±49	211±25	202±35	ns
High-density lipoprotein	43±13	42±8	44±9	ns
Low-density lipoprotein	125±35	135±23	129±31	ns
**Metabolic factors**				
Fasting serum glucose (mg/dl)	95±13	95±15	96±16	ns
Weight (kg)	93±25	96±18	95±19	ns
Body-mass index	32±6	34±5	33±6	ns
Waist circumference (cm)	109±14	108±14	106±14	ns
**Liver histologic findings**				
Total NAFLD activity score	5.7±1.4	5.2±0.8	5.2±1.3	ns
Steatosis	2.1±0.8	1.7±0.8	1.8±0.7	ns
Lobular inflammation	2.1±0.7	1.9±.0.6	1.8±0.6	ns
Hepatocellular ballooning	1.5±0.5	1.6±0.5	1.5±0.5	ns
Fibrosis scores (%)				
Stage 0 or 1	43.7%	66.7%	53.3%	ns
Stage 2	31.3%	26.6%	26.7%	
Stage 3	25%	6.7%	20%	
Stage 4	0	0	0	

### Metabolomic analyses

Using LC/MS and GC/MS analysis, a total of 547 distinct metabolites were identified across the analyzed plasma samples. Of these, 314 biochemicals matched a named structure in the reference library. The remaining 233 represented distinct chemical entities, but they do not match a named biochemical in the reference library.

#### A: Is the histologic response a function of enrichment of plasma with vitamin E?

**Figure 1:**
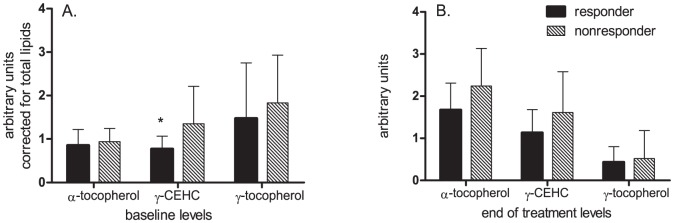
The circulating levels of α-tocopherol, γ-tocopherol and γ-carboxyethylhydroxychroman (CEHC) a metabolite of vitamin E are shown at baseline (panel A) and at end of treatment (panel B) for subjects receiving vitamin E who either met histologic criteria for a response (responders) or met the criteria for non-response. CEHC was significantly lower at baseline in vitamin E treatment responders. The levels of α-, and γ-tocopherols were similar in the two groups at baseline. There was a similar enrichment of α-tocopherol in both responders and non-responders. Conversely, there was a relative de-enrichment of γ-tocopherol in both groups. These data indicate that the histologic response to vitamin E was not a simple response of the amount of vitamin E enrichment of plasma.

To address the possibility that the histologic response was a direct function of the degree of enrichment of plasma with vitamin E, the circulating levels of α-tocopherol, γ-tocopherol and γ-carboxyethyl-hydroxychroman (CEHC) a metabolite of vitamin E were measured at baseline and at the end of treatment [13]. At baseline, the levels of α- and γ-tocopherol were similar and comparable (**Figure 1**). There was a substantial enrichment of the end of treatment plasma with α-tocopherol and γ-CEHC in subjects receiving vitamin E regardless of their histologic response status. As expected, the γ-tocopherol levels were decreased in both these groups. At baseline, CEHC levels were lower in those who responded to vitamin E compared to non-responders (mean: 0.77 vs 1.35 arbitrary units, P = 0.0002). However, the end of treatment histologic response was not related to levels of CEHC, α- or γ-tocopherol. These data indicate that the histologic response to vitamin E was not related to the plasma levels of vitamin E or its metabolites at the end of the study and thus the likelihood of response was not related to compliance alone.

#### B: Identification of predictors of histologic response to vitamin E prior to therapy

This was assessed by comparison of the metabolomic profiles of vitamin E responders vs non-responders at baseline prior to initiation of therapy. Analytes that were significantly different at baseline in responders vs nonresponders were fitted to a logistic regression model to identify those that predicted vitamin_E related histologic response. The significant metabolites identified are listed in **Table 2**. Phenyl-propionic acid and Indole-propionic acid, two intestinal microbiome-derived metabolites [14] were significantly associated with a subsequent histologic response to vitamin E. The levels of asparagine also predicted future response to vitamin E. Conversely, the pre-treatment levels of γ-CEHC a metabolite of vitamin E was inversely associated with response to vitamin E therapy. The levels of γ-palmitoylglycerophosphoethanolamine and myristoleate were also inversely associated with histologic improvement after vitamin E administration.

**Table 2:** Baseline predictors for vitamin E responders.

Metabolites	Odds Ratio (OR) vs non-responders	p-value	95% CI
2-palmitoylglycerophosphoethanolamine	0.08	0.03	0.01–0.56
myristoleate (14∶1n5)	0.04	0.02	0.002–0.64
gamma-CEHC	0.11	0.02	0.01–0.995
Asparagines	20.2	0.01	1.2–338.6
indolepropionate	16.2	0.01	1.45–180.7
3-phenylpropionate (hydrocinnamate)	29.4	0.01	1.23–707.0

Simple logistic regression models were fitted here due to small sample size, significance level is set at α<0.05.

#### C: Differential changes in the metabolome of responders vs non-responders identified glutathione metabolites as potential biomarkers of response

A total of 11 metabolites were differentially expressed between vitamin E responders and vitamin E non-responders at the end of treatment. Two of these were gamma-glutamyl leucine and gamma-glutamyl valine. At baseline, the levels of these compounds were comparable in the two groups. Adjusting for baseline values by ANCOVA, the end of treatment values of these metabolites were significantly lower in vitamin E responders compared to non-responders (**Table 3**). The levels of other metabolites except sphingosine (see below) did not reach significance when adjusted for baseline values. Thus, a histologic response to vitamin E was associated with lower circulating levels of gamma-glutamylated amino acids, indicative of decreased glutathione turnover and improved systemic oxidative stress [14], [15].

**Table 3:** End of treatment vitamin E and placebo response biomarkers.

Vitamin E responder vs Vitamin E non-responder Metabolites	Fold Change	*p*-value
Gammaglutamylleucine	0.82	0.02
Gammaglutamylvaline	0.80	0.03
Sphingosine	0.64	0.02

Placebo responder vs Vitamin E non-responder Metabolites	Fold Change	*p*-value
Gammaglutamylisoleucine	0.67	0.01
Gammaglutamyleucine	0.85	0.05
Sphingosine	0.63	0.02
Gammaglutamyvaline	0.81	0.07

ANCOVA models adjusting for baseline metabolites level with Tukey Method for multiple comparison.

#### D: Relationship of γ-glutamyl peptidase (GGT) levels to histologic response

**Figure 2:**
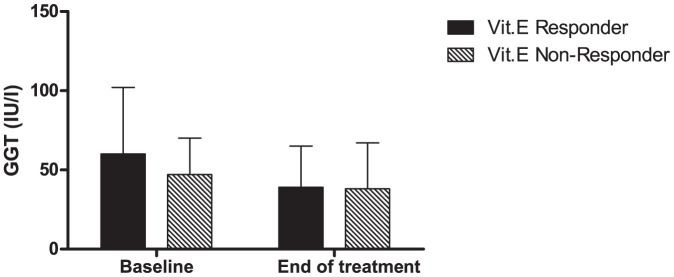
Baseline and end of treatment levels of gamma glutamyl transpeptidase in vitamin E responders and non-responders are shown. The baseline values in both groups are comparable. At the end of treatment, the levels decreased in both groups but the degree of decrease was greater in vitamin E responders. This however was only a trend and not statistically significant.

Under conditions of oxidative stress, intracellular glutathione turnover is increased [16]–[18]. This activates the enzyme γ-glutamyl transpeptidase (GGT) to cleave extracellular glutathione to release gamma-glutamylated amino acids which are taken up for intracellular glutathione reconstitution [16], [18]. Given the relationship of gamma-glutamylated amino acid levels to histologic improvement, we tested the hypothesis that the GGT levels could be used as a surrogate measure of histologic response. The mean GGT levels at baseline were 60 IU/l and 47 IU/l respectively for vitamin E responders vs non-responders (**Figure 2**). While those who had a histologic response and a greater decrease compared to non-responders (mean decrease 21±41 IU vs 9±19 IU, non-significant) this did not reach statistical significance.

#### E. Sphingosine metabolites and response to vitamin E treatment

**Figure 3:**
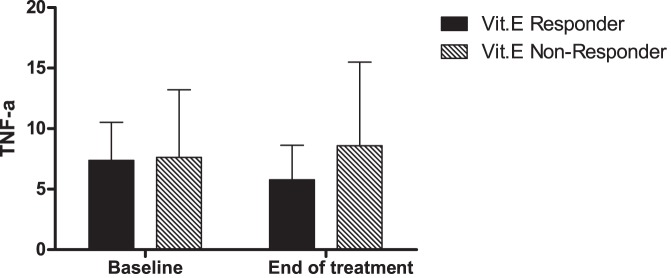
Baseline and end of treatment levels of TNF- α in vitamin E responders and non-responders are shown. The baseline values in both groups are comparable. At the end of treatment, the levels significantly decreased in vitamin E responders (p = 0.002).

A third metabolite whose levels were significantly different in vitamin E responders vs non-responders at end of treatment was sphingosine (**Table 3**). The levels of sphingosine were comparable amongst the two groups at baseline. However, adjusting for baseline levels the end of treatment levels of sphingosine were lower in those with a vitamin E response compared with vitamin E non-responders. TNF-α is a significant predictor for end of treatment sphingosine level after adjusting for baseline sphingosine and treatment group. TNF-α activates sphingomyelinase to release sphingosine. The end of treatment TNF-α is significantly lower in vitamin E responders vs non-responders (**Figure 3**). Other sphingosine-associated metabolites were not significantly different.

#### F: Specificity of metabolic markers of histologic response for vitamin E

This was evaluated from the metabolomic profile of subjects receiving placebo who also achieved histologic improvement (**Table 3**). Adjusting for baseline values by using ANCOVA, placebo-responders also demonstrated levels of gamma-glutamyl leucine and gamma-glutamyl valine that were comparable to vitamin E responders and lower than those seen in vitamin E non-responders. In addition, placebo responders also had a significant decrease in the levels of gamma-glutamyl isoleucine (0.67, *p* = 0.01) and sphingosine (fold change: 0.63, *p* = 0.02). In vitamin E responders, a trend for decreased levels for gamma-glutamyl isoleucine was seen but did not reach significance. When combined with vitamin E treated subjects, gamma-glutamyl leucine and gamma-glutamyl valine remained significant predictors of on-treatment response. These data indicate that histologic response in both those on placebo and in vitamin E responders was associated with a decrease in markers of oxidative stress and intracellular glutathione turnover.

## Discussion

NASH is closely associated with the metabolic syndrome and its related array of biologic disturbances that produces changes in systemic and hepatic metabolism, oxidative stress, apoptosis, inflammatory and fibrotic pathways. These produce changes in the production and utilization of many metabolites in the liver and indeed systemically. The circulating levels of these metabolites reflect an integration of such changes at a systemic level with those in the liver and provide an opportunity to define the “metabolic signature” of NASH at various stages of its natural history and following pharmacologic intervention. The use of such technologies for both type 0 (to identify disease states) and type 1 (to identify therapeutic responses) biomarker (US NIH Biomarkers Definition Group) development is considered a priority in the “liver action plan” for the National Institutes of Health [19], [20]. This exploratory study provides initial evidence that there are measureable differences in the plasma metabolomic profile of (1) thosewho will or will not respond to vitamin E treatment in the future, and (2) those who are improving vs those who are not.

The possibility of avoiding a liver biopsy to assess treatment response is similarly exciting because it removes a barrier towards widespread translation of the results of the PIVENS trial to clinical practice. The realization of this possibility will require confirmation of our data in large scale appropriately designed prospective studies. In this study, we chose the two extreme histologic response phenotypes for comparison i.e. those who met a very robust and tight definition of histologic response and those who had no significant changes in any of the histologic parameters of NASH to provide proof of concept that there are differences in their metabolomic profiles. The metabolomic profiles of those who have some improvement of specific histologic parameters without meeting the primary outcome endpoint of the PIVENS trial are unknown. The potential impact of such “partial histologic responders” on the diagnostic utility of any model developed to assess treatment response to vitamin E also needs additional evaluation.

A key aspect of biomarker development relates to potential pitfalls in the methodology used. Specifically, both biases in treatment assignment and the sequence of sample loading for processing can be a source for potential bias in the data output [9], [12]. These data were collected in the context of the PIVENS trial and the population of subjects assigned to treatment with vitamin E was relatively homogeneous [9]. In our study, special efforts were taken (see methods section) to avoid analytic biases.

The study of metabolomic signatures of subjects on treatment with vitamin E also provides some insights in to the potential effects of vitamin E treatment on the biology of NASH. It is interesting to note that, in an unbiased evaluation of the metabolome, the principal metabolites that distinguished vitamin E responders and non-responders were related to glutathione metabolism. Glutathione is a principal intracellular anti-oxidant and helps maintain redox balance, especially in the liver [14], [15], [21]. Gamma-glutamylated amino acids are established markers of oxidative stress and reflect glutathione turnover [16]. The drop in such amino acids in both those where NASH improves on vitamin E treatment or spontaneously (on placebo) indicates that histologic improvement in NASH under both these conditions is associated with decreased oxidative stress and glutathione turnover. Whether the improvement in oxidative stress improves NASH or vice versa now awaits experimental elucidation.

Sphingosine is released by the action of the tumor necrosis factor-α-sensitive enzyme sphingomyelinase on cell membrane-bound sphingomyelin [22]–[24]. While there is a relative abundance of literature of the potential role of sphingosine-1-phosphate in the metabolic syndrome and its biologic effects, very little is known about the regulation of sphingosine in circulation or its biologic implications. We hypothesize that the novel finding of decreased sphingosine in vitamin E responders reflects decreased TNF-α activity in responders and hope that these findings will provide impetus to future studies of the sphingosine-ceramide pathway in NASH. This is supported by a direct relationship between TNF-α and sphingosine levels in subjects receiving vitamin E; the dependence of sphingosine levels on TNF-α needs to be experimentally verified.

It is also intriguing to note that two of the metabolites (indole-propionic acid and phenyl-propionic acid) that predicted future response to vitamin E therapy can be derived from intestinal microbiota [25]. The tryptophan- and indole-derived compounds have well-established free-radical scavenger and mitochondrial protective properties [26]. These novel findings open the possibility that vitamin E response may depend on the presence of these specific microbiome-derived metabolites or changes in specific microbiota to boost the net anti-oxidant effect achieved.

The principal limitations of this study are the small sample size and the lack of liver tissue for metabolomic analysis which prevent generation of definitive data about biomarkers for response to vitamin E and pathophysiological data about the role of oxidative stress in NASH. However, it is important to emphasize the exploratory nature of this study and that the primary objective was to generate pilot “proof of principle” data to provide direction and support for focused yet large-scale studies to develop biomarkers for response to vitamin E for NASH. By providing such focused data, one may avoid the need for prohibitively expensive large scale full metabolomics profiling to identify such biomarkers.

In summary, this exploratory study provides pilot investigation that there are differences in the circulating profiles of subjects with NASH who will or will not respond to vitamin E treatment and amongst those who are responding to treatment vs those who are not. They also provide several novel findings and insights about the mechanisms of action of vitamin E in NASH which now require further elucidation in specific hypothesis-driven studies. While much work needs to be done to translate these data in to models for personalized clinical care of patients with NASH, this study provides a first step in this direction with respect to the use of vitamin E for NASH.
